# A Novel Technique to Retrieve Cement Retained Implant Supported Prosthesis with Digital Imaging: A Technical Report

**DOI:** 10.30476/dentjods.2024.101241.2285

**Published:** 2025-03-01

**Authors:** Kapil Raju, Fathima Banu Raza, Anand Kumar V

**Affiliations:** 1 Senior Lecturer, Dept. of Prosthodontics, Sri Ramachandra Dental College & Hospital, SRIHER (DU), Porur, Tamil Nadu, India; 2 Reader, Dept. of Prosthodontics, Sri Ramachandra Dental College & Hospital, SRIHER (DU), Porur, Tamil Nadu, India; 3 Professor, Dept. of Prosthodontics, Sri Ramachandra Dental College & Hospital, SRIHER (DU), Porur, Tamil Nadu, India

**Keywords:** Implant-Supported Dental Prosthesis, Computer-Assisted Image Processing, Implant- Abutment connections, 3-Dimensional printing

## Abstract

Retrievability of cement-retained implant-supported prosthesis in the event of abutment screw loosening is a challenging task, and the most common arbitrary drilling technique to retrieve the prosthesis can cause extensive irreversible damage. A 37-year-old male patient reported a chief complaint of a mobile implant-supported prosthesis in the left upper back tooth region. Past dental history revealed that the patient had underwent replacement of missing 27 using cement-retained implant-supported prosthesis 4 years ago using digital impression technique and Computer Aided Designing-Computer Aided Manufacturing (CAD-CAM) milling. This case report describes a novel technique to locate the screw access channel and fabrication of three-dimensional (3D) printed guide template that retrieved the cement-retained prosthesis utilizing the stored digital imaging data. The technique involves designing of the virtual model, screw access channel locator, and prosthesis using CAD technology with the digital impression made during prosthesis fabrication.

## Introduction

Screw-retained prosthesis are commonly indicated in patients with reduced inter-occlusal distance and have the advantage of retrievability and excellent marginal integrity, but complex to fabricate with compromised esthetics, occlusion, and high risk of ceramic fracture due to the presence of screw access hole [ 1 ]. However, the advantages offered by a cement-retained prosthesis such as passive fit, simple laboratory procedures, reduced cost, precise occlusion, and better aesthetics enable clinicians to choose cement-retained prosthesis over screw-retained prosthesis. In the event of unpredictable biological and mechanical failures pertaining to cement-retained implant prosthesis, accessing the abutment screw access channel to retrieve the prosthesis is difficult. The meta-analysis reported by Bozini *et al.* [ 2 ], depicted abutment screw loosening as the most frequent reason for complications in implant treatment with a frequency of 13.4% at the end of 15 years. 

A predictable technique to retrieve the cement-retained prostheses is necessary when screw loosening or abutment fracture occurs. The most commonly used method to locate the screw access channel is arbitrary drilling through the restoration, but this can cause irreversible damage to the abutment screw head and the prosthesis [ 3 ]. With advent of three-dimensional (3D) printing technology and an improvement in material science, this technical report describes a novel technique to locate screw access channel in cement retained implant restorations using digital impression.

## Technique

**Figure 1:**
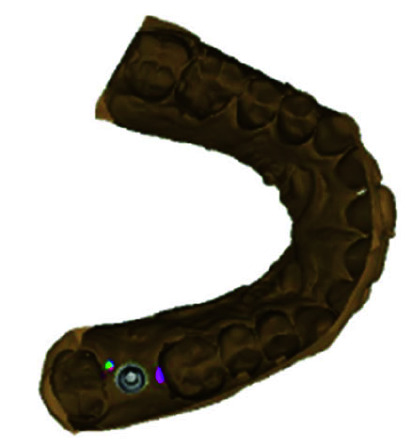
Virtual model designed from the digital impression

**Figure 2:**
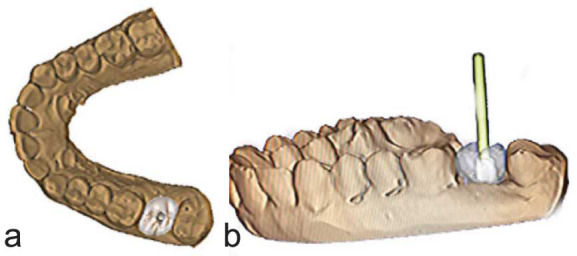
**a:** Virtually designed cement retained prosthesis over the abutment, **b:** Exocad software locating the screw access channel

**Figure 3:**
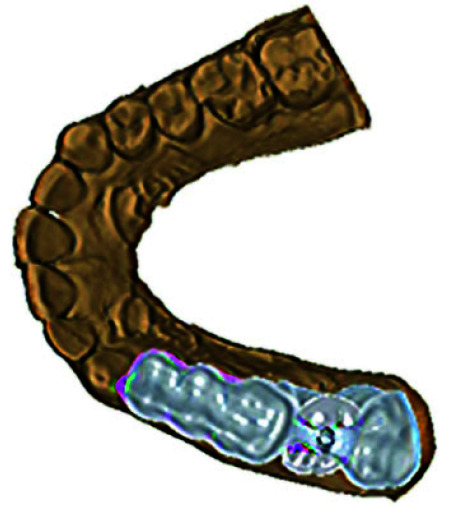
Virtually designed Screw access channel locator guide over the virtual model

**Figure 4:**
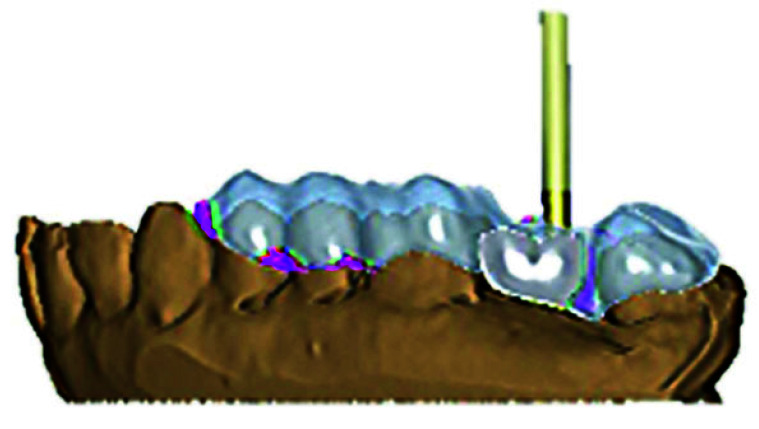
Virtual Screw access channel locator guide indicating abutment screw access using Computer-Aided Designing (CAD)

**Figure 5:**
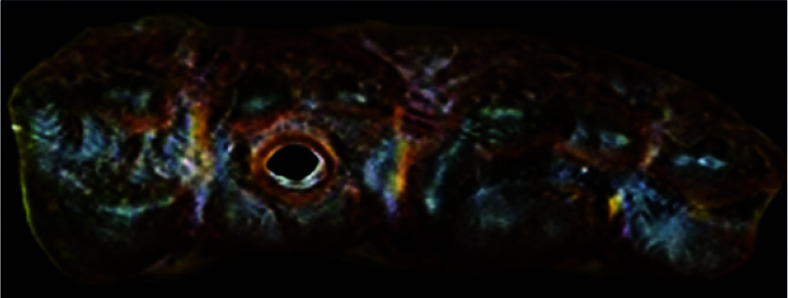
Three-dimensional (3D) printed Screw access channel locator guide

**Figure 6:**
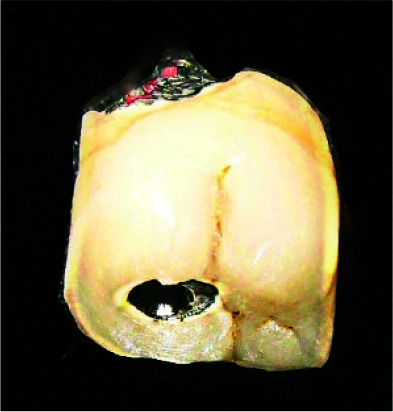
Final access screw opening and retrieved crown

A 37-year-old male patient reported with a chief complaint of crown mobility. Clinical and radiographic examination revealed a healthy implant in 27 tooth region with a cement-retained prosthesis that was mobile depicting abutment screw loosening. On revisiting and retrieval of the patient data, we observed that the patient received a milled prosthesis four years prior with the digitalized impression (TRIOS 3 in 1 Digital Impression Scanner/ TRIOS Color Pod). The abutment screw retrieval for cement-retained implant crown was done by the following steps:

• The scanned digital impression of the abutment (Shining 3D DS-ES Pro) was stored in Surface Tessellation Language (STL) format with patient details (Figure 1).

• The cement-retained implant prosthesis that requires removal was digitally scanned (Medit I700). Both the scanned prosthesis and cone beam computed tomography (CBCT) images were superimposed over the virtual model that was previously stored in STL format (Figure 2a).

• The abutment screw access channel was visualised on the superimposed restoration and the implant from CBCT using the Exocad software (Dental CAD 3.0 Galway) (Figure 2b). This enabled accurate visualisation of the screw access channel.

• The axis guide for the screw access channel was virtually developed on the restoration with a projection of 5 mm to prevent change in the axis of the bur during retrieval (Figure 3).

• The virtual template extending to the adjacent teeth along with the screw access channel locator guide was developed (Figure 4).

• The template was then 3D printed using additive manufacturing technique with the Shining 3D AccuFab-L4D 3D printer (Figure 5).

• The screw channel was accessed intra-orally using the 3D printed screw access channel locator guide and the prosthesis was retrieved (Figure 6). 

## Discussion

The 3D printed method described in this technique to fabricate a screw access channel locator guide assists in the retrieval of cement-retained prosthesis with minimal damage to the prosthesis and abutment. Literature suggests multiple techniques that involve the use of vacuum-formed guides, silicone occlusal index, cylindrical guide hole on the lingual surface of the abutment, photograph to record the position of the abutment before cementation and porcelain stains on the restoration to locate the screw access channel [ 4 - 6 ]. However, these techniques require additional laboratory procedures that need to be planned and fabricated prior to restoration of the implant abutment and the resultant guide needs to be archived for years [ 4 - 6 ]. 

The advent of CAD-CAM and 3D printing technology, the screw access channel can be located precisely which could ease retrieval of the restoration [ 7 ]. Though technological evolution was observed in the literature, the previous technique involved the fabrication of a guide superimposing the scanned images of the metal column indicating the position of the screw access channel and the prosthesis prior to cementation, but this incurred additional chair side time and was cumbersome involving multiple images [ 8 ]. Hence, in our technique, the virtual model, prosthesis, and the guide (screw access channel locator) were designed using CAD technology with the digital impression. CBCT described in the literature for the fabrication of template [ 7 ], can be combined with our technique to identify the screw access channel. However, CBCT should be used with caution because of risk of radiation exposure and also the presence of metallic implant structures can alter the accuracy of the cone bean computed tomography image [ 8 ]. In this technique, the superimposition of CBCT was done to confirm the accuracy of the digital impression scanned data.

The acrylic raw material used for 3D printing (Next Dent Manufacturer) in this technique was industrially polymerized under optimum manufacturing conditions and the additive manufacture yielded precisely fitted 3D printed guides with minimum consumption of the acrylic [ 9 ]. The material helped in maintaining the axis, which are minimally damaged during diamond points drilling the access.

The advantage of this technique is the screw access channel locator guide can be printed on need using the digitally stored data created during prosthesis fabrication and utilized for retrieval of the cement-retained prosthesis. Limitations of this method were the need for storage of preoperative data and the probability of damage to the axis channel during preparation cannot be neglected. However, this method could simplify the abutment retrieval without much damage to the prosthesis.

## Conclusion

Retrievability of cement-retained prosthesis is made easy with a screw access channel locator guide that utilizes the technological advantage of CAD-CAM and 3D printing. A single digitalized record can be utilized for both superstructure and screw access guide fabrication, highlighting the need for digitally storing patient-related clinical data that could be used at the time of retrieval. 
